# Aspirin-Triggered Resolvin D1 (AT-RvD1) Protects Mouse Skin against UVB-Induced Inflammation and Oxidative Stress

**DOI:** 10.3390/molecules28052417

**Published:** 2023-03-06

**Authors:** Cristina P. B. Melo, Priscila Saito, Renata M. Martinez, Larissa Staurengo-Ferrari, Ingrid C. Pinto, Camilla C. A. Rodrigues, Stephanie Badaro-Garcia, Josiane A. Vignoli, Marcela M. Baracat, Allan J. C. Bussmann, Sandra R. Georgetti, Waldiceu A. Verri, Rubia Casagrande

**Affiliations:** 1 Department of Pharmaceutical Sciences, Centre of Health Science, Londrina State University, Londrina 86038-350, PR, Brazil; 2 Department of Pathology, Centre of Biological Sciences, Londrina State University, Rodovia Celso Garcia Cid, Km 380, PR445, Cx. Postal 10.011, Londrina 86057-970, PR, Brazil; 3 Department of Immunology, Harvard Medical School, Blavatnik Institute, Boston, MA 02115, USA; 4 Department of Medicine, Women’s Guild Lung Institute, Cedars-Sinai Medical Center, Los Angeles, CA 90048, USA; 5 Department of Biochemistry and Biotechnology, Centre of Exact Sciences, Londrina State University, Londrina 86057-970, PR, Brazil

**Keywords:** 17(R)RvD1, lipid mediator, ROS, Nrf2, ciclooxygenase-2, cytokines, ultraviolet radiation, antioxidant capacity, neutrophil infiltration, sunburn

## Abstract

Intense exposure to UVB radiation incites excessive production of reactive oxygen species (ROS) and inflammation. The resolution of inflammation is an active process orchestrated by a family of lipid molecules that includes AT-RvD1, a specialized proresolving lipid mediator (SPM). AT-RvD1 is derived from omega-3, which presents anti-inflammatory activity and reduces oxidative stress markers. The present work aims to investigate the protective effect of AT-RvD1 on UVB-induced inflammation and oxidative stress in hairless mice. Animals were first treated with 30, 100, and 300 pg/animal AT-RvD1 (i.v.) and then exposed to UVB (4.14 J/cm^2^). The results showed that 300 pg/animal of AT-RvD1 could restrict skin edema, neutrophil and mast cell infiltration, COX-2 mRNA expression, cytokine release, and MMP-9 activity and restore skin antioxidant capacity as per FRAP and ABTS assays and control O_2_^•−^ production, lipoperoxidation, epidermal thickening, and sunburn cells development. AT-RvD1 could reverse the UVB-induced downregulation of Nrf2 and its downstream targets GSH, catalase, and NOQ-1. Our results suggest that by upregulating the Nrf2 pathway, AT-RvD1 promotes the expression of ARE genes, restoring the skin’s natural antioxidant defense against UVB exposition to avoid oxidative stress, inflammation, and tissue damage.

## 1. Introduction

UVB is the most energetic ultraviolet radiation fraction from the sun to reach Earth. It penetrates the epidermis and, in high doses, induces sunburn, an acute inflammatory reaction that is clinically characterized by painful erythema, heat, and swelling [1]. UVB is harmful to cells affecting, for instance, the cellular metabolism by disrupting regulatory pathways and causing morphological and structural changes and DNA damage. All these factors play together to promote and initiate nonmelanoma skin cancer, one of the most frequently diagnosed types of cutaneous malignancies induced by UVB [2].

Acute inflammation is a physiological response aiming to protect the organism against noxious agents and tissue injury. In normal conditions, this process is self-contained to allow resolution towards a homeostatic state [3]. It is already common knowledge that the resolution of inflammation is an active process, regulated by biochemical mediators and receptor-signaling pathways, which is driven by specialized proresolving lipid mediators (SPMs), such as lipoxins, E-series resolvins, D-series resolvins, protectins/neuroprotectins, and maresins [4]. Resolvins of the D series (RvD) are metabolites of a 22-carbon polyunsaturated fatty acid (PUFA), the docosahexaenoic acid (DHA) [5,6]. They follow two distinct biosynthetic pathways: one leads to the production of 17 (S)-hydroxylated D-resolvin or RvD1 and relies on the enzyme cyclooxygenase-2 (COX-2) and the other delivers 17 (R)-hydroxylated D-resolvin or aspirin-triggered RvD (AT-RvD; 17(R)RvD1; 7S,8R,17R-trihydroxy-4Z,9E,11E,13Z,15E,19Z-docosahexaenoic acid), which is the product of aspirin-acetylated-COX-2 [7,8].

AT-RvD1 presents strong anti-inflammatory and immunoregulatory properties [9]. It exhibits potent anti-inflammatory action in vivo [10] and is reported to limit polymorphonuclear (PMN) infiltration into the brain, skin, and peritoneum and to downregulate the extracellular release of superoxide anion (O_2_^•−^) by neutrophils [11]. Furthermore, AT-RvD1 shows marked analgesic effects in acute inflammation in adjuvant-induced arthritis in rats [12], carrageenan paw inflammation, and monosodium iodoacetate-induced osteoarthritis [13]; prevents colitis in two different models of intestinal inflammation [14]; and decreases inflammation and oxidative stress markers and ROS production after cigarette smoke-induced emphysema [15].

Studies have been focusing on the treatment of acute skin damage caused by UVB using antioxidants and inhibitors of the inflammation cascade [16,17,18,19,20,21,22]. A novel approach would be to investigate the therapeutic potential of resolution inducers against UVB-induced inflammation and oxidative stress [23,24,25]. As far as we know, only one study has been conducted to investigate the effects of a resolvin, specifically RvD1, in UVB inflammation and oxidative damage to the skin [26]. Nevertheless, evidence supports that AT-RvD1 has a higher potency than RvD1 in reducing inflammation. For instance, RvD1 reduces the LPS-induced production of TNFα by human peripheral blood mononuclear cells by 37% and AT-RvD1 reaches 69% inhibition at the same concentration. AT-RvD1 also presented significant inhibition against the production of other cytokines such as IL-7, IL-12, IL-8, CCL2, and MIP-1α, which was not significantly affected by treatment with the same concentration of RvD1 [27]. In ovalbumin antigen-challenged asthma-like pulmonary inflammation, AT-RvD1 presented a more prominent activity than RvD1 against the recruitment of eosinophils and almost doubled the speed of inflammation resolution compared to RvD1. Thus, AT-RvD1 reduced pulmonary inflammation more robustly than RvD1 with the same treatment regimen [28]. Therefore, AT-RvD1 and RvD1, although with slightly different structures, have significant activity differences, and AT-RvD1 might be active at lower doses or reach higher efficacy than RvD1. Considering this scenario in which AT-RvD1 could be potentially more promising than RvD1 due to the activity in lower doses, we reasoned that it was necessary to investigate the activity of AT-RvD1 in UVB-triggered skin inflammation and oxidative stress and pursued this aim in the present study.

## 2. Results

In this work, the UVB dose of 4.14 J/cm^2^ was sufficient to trigger the effects of UVB exposure on acute inflammation parameters. This dose was chosen based on results from prior standardization studies in which the same dose showed results consistent with the human inflammatory response from acute UVB stimulation [29,30,31]. The administration route was chosen based on results obtained from earlier studies using AT-RvD1 with diverse disease models [14,32]. Three doses were selected, based on preliminary results obtained by our group (data not shown). Inflammatory and oxidative parameters, such as edema, neutrophil infiltration (such as MPO activity), and zymography showing MMP-9 activity, along with skin antioxidant capacity (measured by FRAP and ABTS) and GSH level, were measured to select the best dose of AT-RvD1. All these parameters peak at 12 h after UVB radiation; thus, in these experiments, we performed a dose–response. All the remaining tests were performed with the selected dose of 300 pg/mouse.

### 2.1. Edema and Neutrophil Infiltration

**Figure 1:**
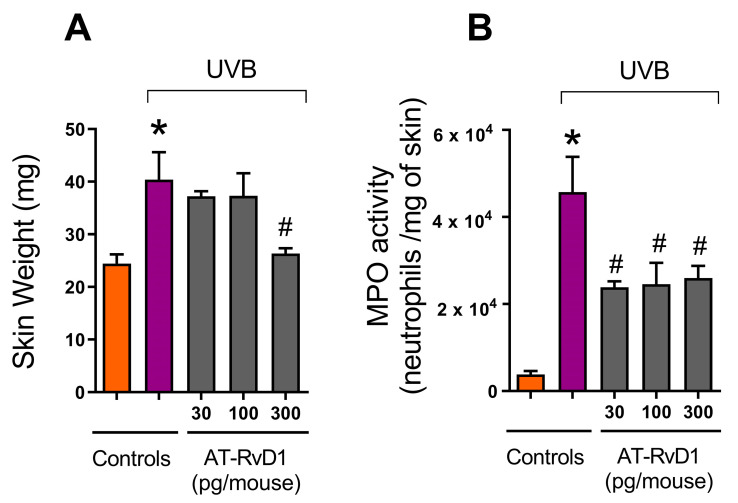
Effect of three doses of AT-RvD1 on edema and neutrophil infiltration in the skin of hairless mice exposed to 4.14 J/cm^2^ UVB radiation. Edema (**A**) and neutrophil infiltration (quantitated by the MPO activity) (**B**). Skin samples were collected 12 h after the radiation session. Bars represent means ± SEM. Two separate experiments with five groups of 5 mice per group per experiment were performed. Statistical analysis was performed by one-way ANOVA, followed by Tukey’s post hoc test. * *p* < 0.05 compared to the nonradiated control group (orange bars); # *p* < 0.05 compared to the radiated control group (purple bars).

Edema is an early marker of UVB-induced acute inflammation [33]. In this work, the weights of standard-sized skin samples obtained from mice treated with 30, 100, and 300 pg of AT-RvD1 per animal, were compared to controls (naive and UVB) (Figure 1A). UVB controls showed a significant increase in skin weight compared to naive. The samples from 30 and 100 pg of AT-RvD1 followed the same trend (Figure 1A), but the samples from mice treated with 300 pg/mouse weighed significantly less, being equivalent to naive (Figure 1A), indicating a more pronounced reduction of skin edema. Furthermore, UVB control samples presented considerably higher MPO activity, while all three doses of AT-RvD1 were able to control this effect (Figure 1B). Because MPO activity is an indicator of neutrophil infiltration [34], it can be concluded that UVB radiation increased the accumulation of neutrophils in the skin while the three doses of AT-RvD1 controlled the influx of these cells to the tissue. Considering that the dose of 300 pg/mouse of AT-RvD1 was the only one to inhibit both skin edema and MPO activity, it was selected for the following experiments in which sample collection occurred 2 or 4 h after UVB radiation.

### 2.2. Epidermal Thickness and Sunburn Cells

**Figure 2:**
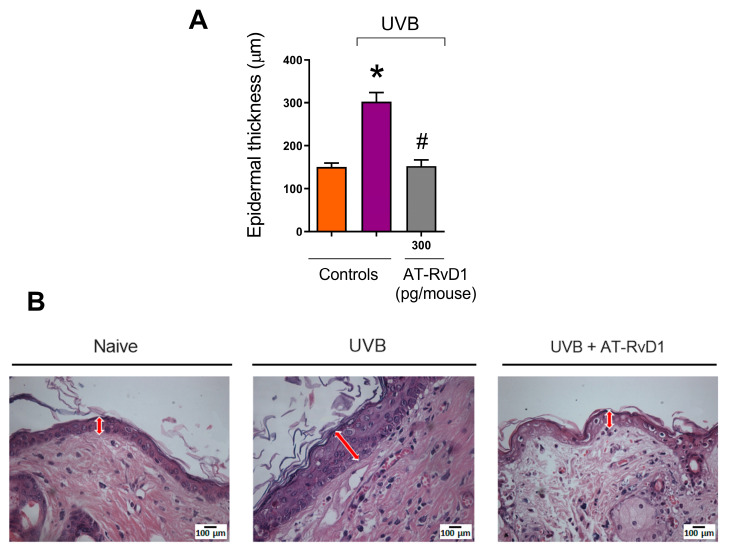
Effect of AT-RvD1 on epidermal hyperplasia of hairless mice exposed to 4.14 J/cm^2^ UVB radiation. Epidermal thickness (µm) (**A**) and representative images of the epidermal thickness of the three tested groups—H&E stain (**B**). Skin samples were collected 12 h after the exposure. Arrows indicate representative epidermal thickness. Bars represent means ± SEM. Two separate experiments with five groups of 5 mice per group per experiment were performed. Statistical analysis was performed by one-way ANOVA, followed by Tukey´s post hoc test. * *p* < 0.05 compared to the nonradiated control group (orange bar); # *p* < 0.05 compared to the radiated control group (purple bar).

Exposure to UVB radiation promotes epidermal hyperplasia, causing skin thickening, a common reaction to excessive UVB exposure [35]. On the other hand, the samples from mice treated with 300 pg/mouse AT-RvD1 did not present a hyperplastic epidermis (Figure 2A,B).

**Figure 3:**
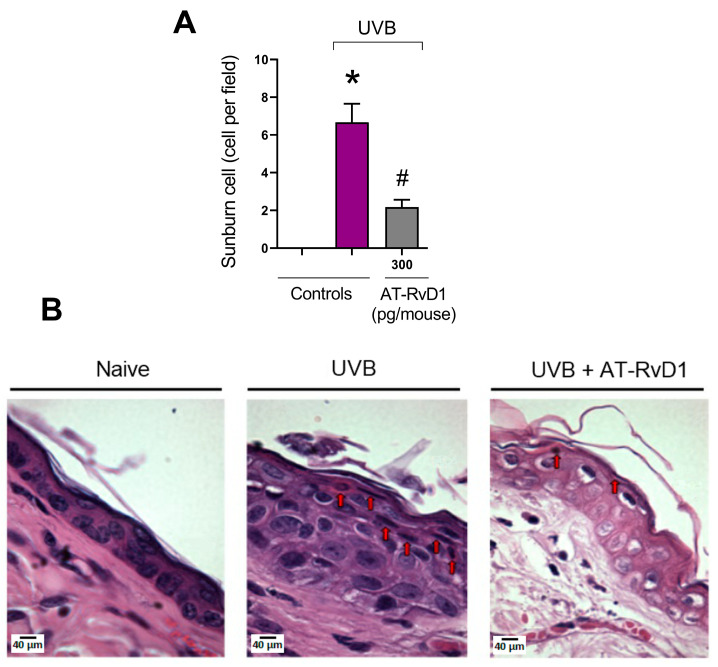
Effect of AT-RvD1 on the number of epidermal sunburn cells in hairless mice exposed to 4.14 J/cm^2^ UVB radiation. The number of sunburn cells (**A**) and representative images of sunburn cells in the three tested groups—H&E stain (**B**). Skin samples were collected 12 h after the exposure. Arrows indicate examples of sunburn cells. Bars represent means ± SEM. Two separate experiments with five groups of 5 mice per group per experiment were performed. Statistical analysis was performed by one-way ANOVA, followed by Tukey´s post hoc test. * *p* < 0.05 compared to the nonradiated control group (orange bar); # *p* < 0.05 compared to the radiated control group (purple bar).

Sunburn cells can be identified morphologically and biologically as keratinocytes undergoing apoptosis and are a characteristic cell type of skin exposed to UV radiation upon doses around or above the minimum erythemal dose [36]. In this work, the group treated with 300 pg/mouse of AT-RvD1 presented a significantly lower number of sunburn cells when compared to the UVB control (Figure 3).

### 2.3. Dermal Mast Cell Count

**Figure 4:**
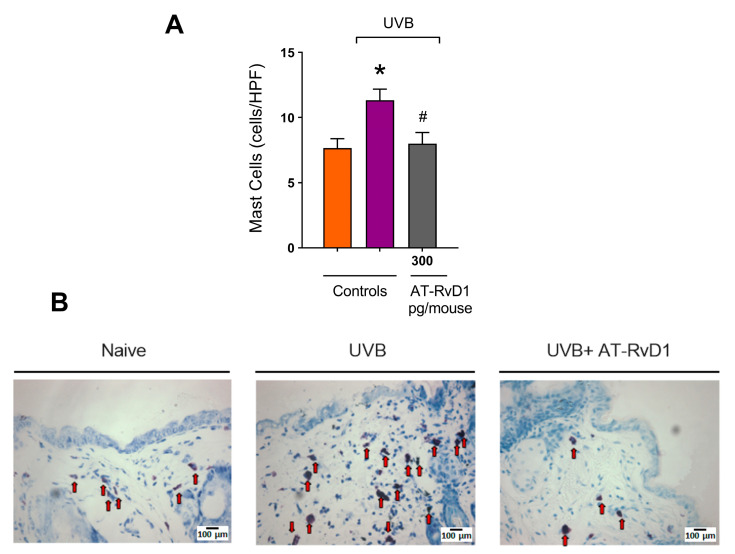
Effect of 300 pg/mouse AT-RvD1 on dermal mast cell infiltration in hairless mouse skin exposed to 4.14 J/cm^2^ UVB radiation. The number of mast cells (**A**) and representative images of mast cells in the toluidine-blue-stained dermis (**B**). Skin samples were collected 12 h after the exposure. Arrows indicate mast cells. Bars represent means ± SEM. Two separate experiments with five groups of 5 mice per group per experiment were performed. Statistical analysis was performed by one-way ANOVA, followed by Tukey´s post hoc test. * *p* < 0.05 compared to the nonradiated control group (orange bar); # *p* < 0.05 compared to the radiated control group (purple bar).

Mast cells are typical tissue resident cells in the dermis, but UVB radiation enhances their number and promotes physical changes such as hypogranulation due to degranulation [37]. A rise in the mast cell number was observed in the UVB control group compared to the naive group. In contrast, in mice treated with 300 pg/mouse AT-RvD1 before UVB exposure, this number was maintained within basal levels (Figure 4A,B).

### 2.4. MMP-9 and Collagen Fiber Degradation

**Figure 5:**
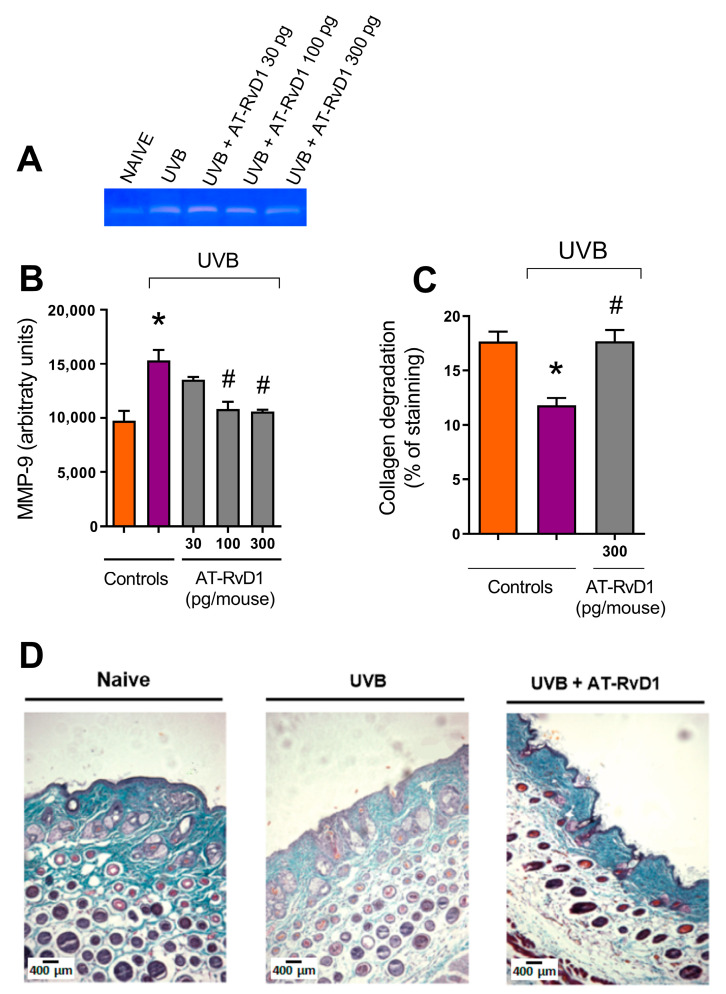
Effect of 300 pg/mouse AT-RvD1 on the increase of MMP-9 activity and related collagen damage in hairless mouse skin exposed to 4.14 J/cm^2^ UVB radiation. Skin samples were collected 12 h after the exposure. Representative image of acrylamide-SDS-gelatin zymography gel showing discolored zones that represent MMP-9 activity (**A**); MMP-9 activity per group (**B**); collagen fiber intensity evaluated as a percentage of staining, compared per group (**C**); and representative image of collagen fiber intensity and bundles, shown in blue, of the three tested groups (**D**). Images in panel (**D**) were analyzed and quantitated using the Image J software, version 1.51 (U. S. National Institutes of Health, Bethesda, MD, USA) to generate the results shown in panel (**C**). Bars represent means ± SEM. Panel (**B**) presents the results of four pools of the dorsal skin of two mice per pool. For panels (**C**,**D**), two separate experiments with five groups of 5 mice per group per experiment were performed. Statistical analysis was performed by one-way ANOVA, followed by Tukey´s post hoc test. * *p* < 0.05 compared to the nonradiated control group (orange bars); # *p* < 0.05 compared to the radiated control group (purple bars).

UVB radiation enhances the activity of MMP-9, thus intensifying collagen fiber degradation [38,39]. Treatments with 100 and 300 pg/mouse of AT-RvD1 controlled this effect. Considering that 300 pg/mouse was the only dose that demonstrated efficiency on a broad set of tests, it was the treatment of choice for histological evaluation and was demonstrated to also be effective against collagen degradation (Figure 5).

### 2.5. COX-2 Expression

**Figure 6:**
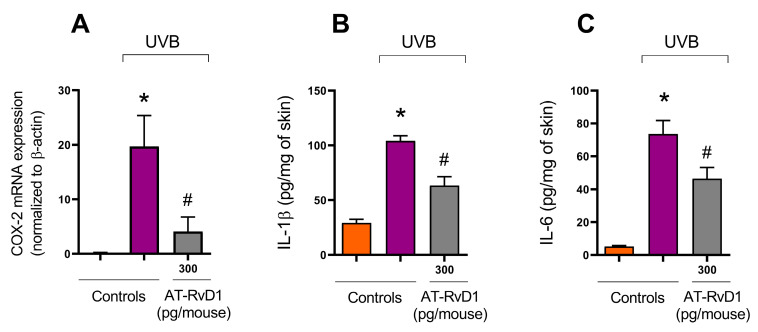
Effect of 300 pg/mouse AT-RvD1 on COX-2 mRNA expression determined using reverse transcriptase quantitative polymerase chain reaction (RT-qPCR) and on cytokine levels determined using ELISA in hairless mouse skin exposed to 4.14 J/cm^2^ UVB radiation. Skin samples for the determination of COX-2 mRNA expression and for cytokine levels were collected 4 h after UVB exposure. COX-2 mRNA expression (**A**); IL-1ꞵ (**B**); IL-6 (**C**). Bars represent means ± SEM. Two separate experiments with five groups of 5 mice per group per experiment were performed. Statistical analysis was performed by one-way ANOVA, followed by Tukey´s post hoc test. * *p* < 0.05 compared to the nonradiated control group (orange bars); # *p* < 0.05 compared to the radiated control group (purple bars).

COX-2 is a proinflammatory enzyme whose expression is enhanced in inflammation and is found in polymorphonuclear leukocytes [40]. Acute exposure to UVB radiation enhances the enzyme’s activity in the skin. Results from a RT-qPCR showed a significant increase in mRNA expression for COX-2 in skin samples collected 4 h after UVB irradiation (Figure 6A). Samples collected under the same conditions from the group radiated and treated with 300 pg/mouse of AT-RvD1 presented a 4.8-fold reduction in the expression of mRNA for COX-2 in comparison to the UVB control group (Figure 6).

### 2.6. Cytokine Levels

UVB radiation is a trigger to cytokine production [41,42], which is demonstrated by the significant rise in the levels of all tested cytokines in the UVB control group compared to the naive group. Nevertheless, the treatment with 300 pg/mouse of AT-Rv-D1 limited the excessive release of all tested cytokines (Figure 6B,C).

### 2.7. Skin Redox Balance

**Figure 7:**
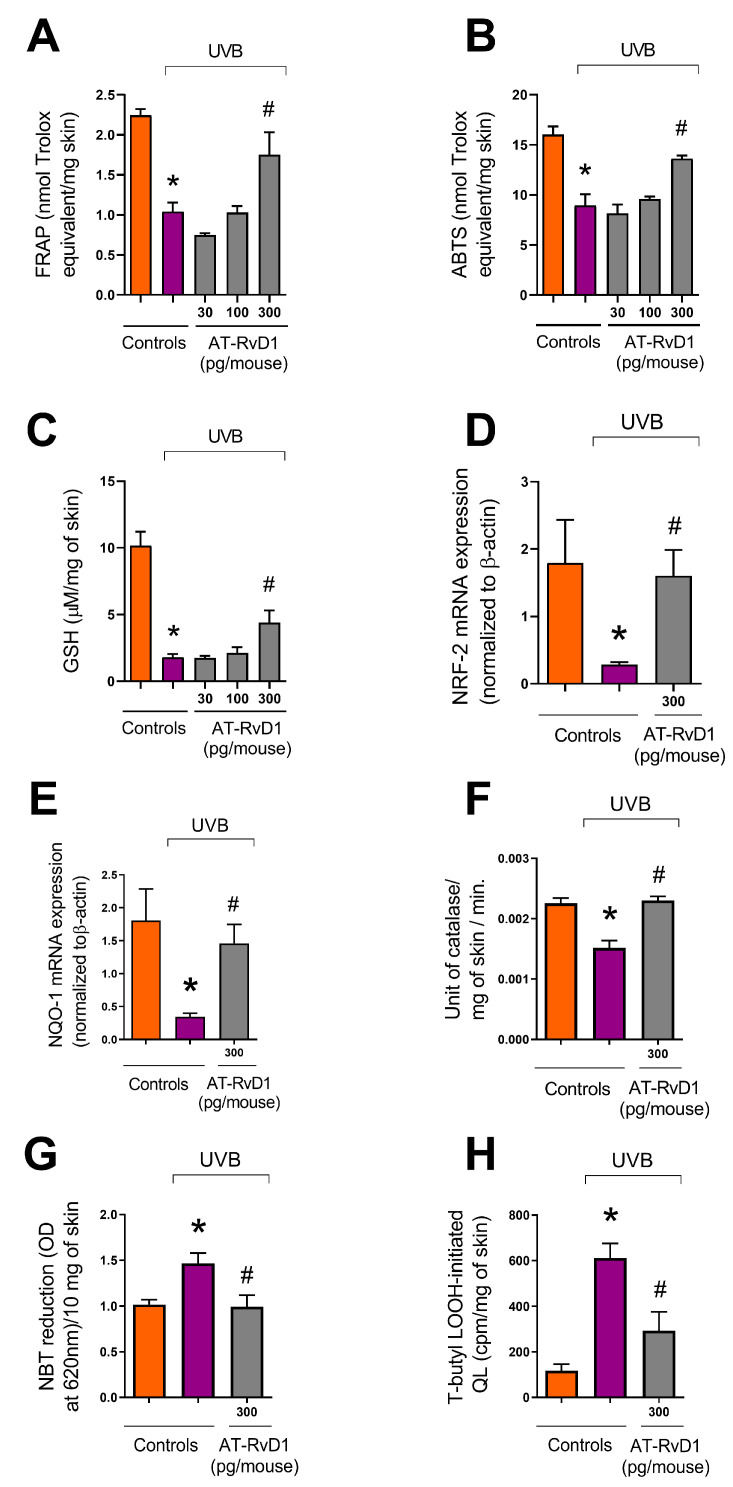
Effect of AT-RvD1 on the skin’s natural antioxidant capacity in hairless mice exposed to 4.14 J/cm^2^ UVB radiation. Ferric Reducing Antioxidant Power (FRAP) (**A**); scavenging capacity of ABTS+ (**B**); GSH level (**C**); Nrf2 mRNA expression (**D**); NQO-1 mRNA expression (**E**); catalase activity (**F**); O_2_^•−^ formation measured by NBT reduction to formazan (**G**); lipid peroxidation (**H**). Time points for collecting skin samples were: 12 h after UVB exposure for FRAP, ABTS, and GSH; 4 h after exposure for Nrf2 and NQO-1 mRNA expression and lipid peroxidation; and 2 h after exposure for catalase activity and O_2_^•−^ formation. Bars represent means ± SEM. Two separate experiments with five groups of 5 mice per group per experiment were performed. Statistical analysis was performed by one-way ANOVA, followed by Tukey´s post hoc test. * *p* < 0.05 compared to the nonradiated control group (orange bars); # *p* < 0.05 compared to the radiated control group (purple bars).

Data showed that UVB radiation leads to the overproduction of ROS and depletion of the skin’s endogenous antioxidant capacity. FRAP was significantly lowered in the UVB control group compared to the naive group (Figure 7A). The same profile was observed for the ABTS+ scavenging capacity (Figure 7B). Treatment with 300 pg/mouse of AT-RvD1 restored the skin’s antioxidant capacity to the same levels found in the naive group (Figure 7A,B). The results also showed that the UVB control group had a significant decrease in GSH levels and that this effect could be controlled by the treatment with 300 pg/mouse of AT-RvD1 (Figure 7C).

Because the endogenous antioxidant capacity relies on an intricate network of reactions, several methods are needed to evaluate the effects of UVB exposition on the skin’s redox state and the efficiency of the treatment with AT-RvD1 to prevent oxidative stress [43,44]. UVB radiation promoted depletion of the erythroid nuclear factor (Nrf2) with a consequent reduction of NAD(P)H quinone oxidoreductase-1 (NQO-1) and catalase, both cytoprotective enzymes induced by Nrf2. Treatment with AT-RvD1 restored Nrf2 and NQO-1 mRNA expression, as well as catalase activity (Figure 7C–E). UVB radiation also elevated the production of the superoxide anion (O_2_^•−^) as measured by the reduction of NBT to formazan. Treatment with AT-RvD1 kept O_2_^•−^ at basal levels (Figure 7F). Lipid peroxidation is a crucial step in the sequence of reactions that culminate in oxidative stress [45]. Simultaneously, the lipid membrane is both a target and a source of free radicals, promoting a cycle loop of ROS formation [46]. It was observed that UVB radiation led to an increase in the formation of lipid peroxides, while treatment with AT-RvD1 could control this effect (Figure 7H). Therefore, it is likely that triggering antioxidant responses via induction of Nrf2 and its downstream targets NQO-1, catalase, and GSH might explain the AT-RvD1 biological activity against UVB deleterious effects in the skin.

## 3. Discussion

Resolution of inflammation is an active and coordinated process characterized by molecular and cellular events aimed to ensure tissue repair and the regaining of physiological function to prevent the progression from acute to persistent chronic inflammation [47]. The switch from inflammation initiation to resolution occurs at the cellular level and is marked by events that include reduction of neutrophil infiltration, neutrophil apoptosis, and efferocytosis that also switches the macrophage phenotype. Changing the metabolization of ω6 and ω3 fat acids from proinflammatory lipid molecules towards SPMs seems to be an essential step to achieve inflammation resolution [48,49]. In addition to the endogenous roles of SPMs, they can per se be used as pharmacological therapies to reduce inflammation and initiate resolution. AT-RvD1 is an SPM derived from docosahexaenoic acid and classified as a D series resolvin produced by aspirin-acetylated COX-2. AT-RvD1 is active in lower doses/concentrations compared to RvD1 [27,28], which supports that it would have a better profile as a druggable molecule since the costs would be lower as well. In a prior study, AT-RvD1 proved to be statistically more potent than RvD1 at the same dose when taking as a parameter for comparison the decrease of leukocyte infiltration in a model of murine peritonitis. Additionally, in the same study, AT-RvD1 demonstrated more resistance than RvD1 to catalysis by eicosanoid oxidoreductases in vitro. The authors proposed that AT-RvD1 resistance to metabolization may explain its higher efficiency in comparison with RvD1 [3]. The present study evaluated the protective effects of systemic treatment with AT-RvD1 on UVB-induced inflammation and oxidative stress in mice.

It is well established that UVB radiation induces sunburn, an acute inflammatory response in the skin, which is characterized by the appearance of erythema and edema promoted by vasodilation in the dermis, accompanied by epidermal and dermal neutrophil infiltration, followed by macrophage influx [1,50,51]. AT-RvD1 showed the capacity to control neutrophil infiltration and promote neutrophil clearance both in vivo and in vitro in different organs and models of inflammation including skin, brain, uterus, intestines, and joints [3,11,12,14,15,48,52]. Our results are in line with those previous data since even the smallest dose administered of AT-RvD1 (30 pg/animal) could significantly reduce the neutrophil infiltration promoted by UVB stimulation. Because neutrophils produce and release proinflammatory substances such as cytokines, ROS, and proteolytic enzymes that are harmful to cells and tissues [51,53], by controlling neutrophil infiltration, AT-RvD1 might prevent the aggravation of the inflammatory process and have a cytoprotective role.

The protective effect of AT-RvD1 is even more evident in the results from epidermal thickness, in which, 12 h after UVB exposure, samples from mice pretreated with 300 pg/mouse of AT-RvD1 showed significantly less thickening than those from the UVB-stimulated group. Along with melanogenesis, hyperplasia of epidermal cells is a characteristic defense reaction of the skin against UVB-induced damage to macromolecules, especially DNA [35,54]. The rise in the number of sunburn cells is also a marker of UVB-generated skin damage. Sunburn cells are keratinocytes that undergo apoptosis when the UVB irradiation surpasses their protective-response threshold. These cells represent a protection mechanism that targets the elimination of DNA-damaged cells. In the epidermis, they appear after 30 min and peak at 24 h after UVB exposure and are identified by their shrunken chromatin and eosinophilic cytoplasm [55,56]. Earlier findings suggest that apoptosis induced by UVB is, at least in part, mediated by the p53/p21/Bax/Bcl-2 pathway. It has also been demonstrated that dead cells are replaced by hyperproliferative ones, leading to epidermal hyperplasia. UV-induced apoptosis and hyperplasia seem to be tightly linked and the deregulation of these pathways may contribute to cancer development [16,56,57]. In this sense, the significant reduction of sunburn cells in samples from animals treated with AT-RvD1 highlights the therapeutic potential of the drug.

Mast cells are tissue-resident cells in the dermis, but it has been demonstrated, in the present and in prior studies, that UVB irradiation increases their number and changes their profile. In fact, samples submitted to irradiation show a higher number of degranulated mast cells [25,26,43]. Mast cells release various inflammatory mediators, such as histamine, interleukins, and leukotrienes, which are implicated in edema and neutrophil accumulation at the inflammation site [58]. Histamine induces PGE_2_ production by keratinocytes; influences the migration of immune cells to UVB-radiated sites; and regulates their proliferation and cytokine profile. Furthermore, histamine is also one of the mediators involved in the immunosuppression that can be detected after repetitive and long-term exposure to UVB [59,60]. Our results show that the increased number of mast cells correlates well with the increase in COX-2 mRNA expression and the release of cytokines in the UVB-stimulated group, while these effects were prevented in the group treated with AT-RvD1. Hence, the literature shows that AT-RvD1 controls histamine-stimulated responses in rat goblet cells by preventing the increase in Ca^2+^ and activation of ERK1/2 by histamine activation of its H1 receptor subtype [61].

Treatment with AT-RvD1 could also inhibit UVB radiation-induced MMP-9 activity increase and prevent damage to the dermal collagen matrix. MMP-9 is a collagenase that belongs to the group of MMPs and is specifically responsible for degrading collagen IV. MMPs are implicated in the degradation of the fiber network and collagen matrix in the dermis, so much so that MMP-induced damage to the collagen is a hallmark of photoaging and skin cancer [62,63]. Since neutrophils, keratinocytes, mast cells, and endothelial cells produce MMP [64,65], and because ROS are also implicated in their overexpression via protein kinase cascade [51], our results showcase the ample benefits of AT-RvD1 mechanisms in blocking the network of inflammatory and oxidative events that lead to damage of the dermal matrix and further aggravation of inflammation

Keratinocytes and immune cells release various proinflammatory cytokines upon UVB radiation. In the present study, IL-1ꞵ and IL-6 were substantially increased after UVB irradiation, but treatment with AT-RvD1 could control this effect, maintaining cytokine levels close to those found in the naive group. The efficacy of AT-RvD1 in this context can be confirmed by its ability to control the progression of events that are influenced by the production of these cytokines: i.e., compared to the UVB group, the AT-RvD1 treated group showed milder edema; reduced neutrophil and mast cell infiltration; less collagen degradation, which is a result of controlled MMP-9 activation; fewer sunburn cells; and less epidermal thickening. Neutrophil recruitment, metalloproteinases, and ROS production are increased by cytokine release [41]. IL-1ꞵ, for instance, intensifies ROS production through increased NADPH oxidase expression and activates metalloproteinases through ROS and MAPK pathways [19,51]. IL-6 is implicated in promoting leukocyte infiltration and sunburn reaction. Corroborating our results, AT-RvD1 also significantly decreased the levels of proinflammatory cytokines, including IL-1β and IL-6 [52]. AT-RvD1 caused pronounced and long-lasting analgesic effects in a model of adjuvant-induced arthritis in rats through inhibition of proinflammatory and pronociceptive mediators, including IL-1β, and through the blockage of COX-2 mRNA expression and NF-kB activation [12].

The results from the edema assay demonstrate that treatment with AT-RvD1 (specifically 300 pg/mouse) could prevent UVB-induced swelling of the tissue and are in line with the results obtained from the RT-qPCR showing a reduction of approximately 4.8-fold in COX-2 mRNA expression in the treated group compared to the UVB-stimulation group. UVB induces COX-2 expression in keratinocytes via EGFR activation, which is also responsible for the upregulation of IL-8 and for TNF-α gene expression [66]. Through the COX-2 pathway, PGE_2_ is abundantly produced by keratinocytes and plays a crucial role in vasodilation and erythema after UVB exposure [67]. In line with our results, an earlier study demonstrated that systemically delivered AT-RvD1 could decrease vascular permeability and neutrophil infiltration in endometriosis in vivo and also that the treatment could dose-dependently reduce neutrophil migration across the endothelial layer in vitro [32].

Oxidative stress is an important factor in the pathology of UVB-induced inflammation. UV photons interacting with endogenous photosensitizers in the skin promote the generation of ROS such as O_2_^•−^, which through superoxide dismutase (SOD) activity generates hydrogen peroxide (H_2_O_2_). Finally, the powerful oxidant hydroxyl radical (HO^•^) is produced by the oxidation of H_2_O_2_ in the presence of redox-active transition metals [54,68,69]. Excessive ROS production and insufficient scavenging lead to biomolecule oxidation, cell structure and function alterations, DNA mutations, and dysregulation of cell signaling pathways. Furthermore, as mentioned before, oxidative stress can compromise the resolution timeline by supporting the intensification of the inflammatory process, leading to a chronic inflammatory state, which is a common feature of many skin diseases [70,71]. In the present work, the excessive O_2_^•−^ production displayed by the UVB-stimulated group can be explained not only by UVB’s high capacity of energy transfer but also by the intense accumulation of neutrophils in the tissue, which is confirmed by the MPO results (Figure 1B). UV-irradiated neutrophils have a high potential for ROS production and often release their content of granules into the extracellular matrix [72]. Our results show that the AT-RvD1-treated group presented basal levels of O_2_^•−^ comparable to naive controls.

HO^•^ and hydroperoxyl (HO_2_^•^, a protonated form of O_2_^•−^) are important ROS in promoting polyunsaturated phospholipids peroxidation, an oxidative chain reaction that promotes membrane dysfunction and generates highly reactive aldehydes such as malondialdehyde (MDA), propanal, hexanal, and 4-hydroxynonenal (4-HNE). Among those reactive aldehydes, MDA is the most mutagenic [73]. MDA can modify a series of molecules involved in humoral defense, the respiratory chain, energy metabolism, neuronal communication, the matrix, structural proteins, proteolytic system, and endogenous antioxidants [46]. The skin’s antioxidant defense is a robust and interactive network of both chemical and enzymatic antioxidants aimed to protect the organ from ROS damaging effects. Ascorbic acid, tocopherol, uric acid, and reduced glutathione (GSH) are a few examples of chemical antioxidants produced by the skin. Enzymatic antioxidants such as catalase, superoxide dismutase (SOD), glutathione peroxidase (GPx), and glutathione reductase (GR) also play a role in the skin’s antioxidant defense [68,69]. We focused our research on GSH and the enzyme catalase. Results from the UVB-exposed group demonstrated that the high production of ROS led to oxidative stress, as highlighted by the lowered antioxidant capacity of the skin and the lower levels of GSH in this group. Antioxidant capacity measured by FRAP is related to tissue levels of molecular antioxidants such as ascorbic acid, uric acid, and α-tocopherol [74], while results from ABTS are linked to GSH levels [75]. The oligopeptide GSH is a primary antioxidant of the scavenging system that functions as an electron donor to ROS and other highly reactive molecules. Elevation of GSH levels is considered an early defense response to oxidative stress and its depletion is associated with high production of O_2_^•−^, the first in a cascade of UVR-generated ROS. O_2_^•−^ is reduced to H_2_O_2_ by SOD, and subsequently reduced to H_2_O by glutathione peroxidase with the aid of GSH, which is oxidized to GSSG during the operation. To restore the antioxidant capacity, GSSG must be reduced back to GSH by glutathione reductase using NADPH as an electron donor. Therefore, depletion of GSH works as an efficient indicator that the scavenging and antioxidant mechanisms of the tissue are overwhelmed by the intense production of ROS, which means that oxidative stress is installed [70]. As our results indicate, treatment with 300 pg/mouse of AT-RvD1 preserved the antioxidant capacity in UVB-irradiated skin by maintaining FRAP levels, ABTS scavenging capacity, and restoring GSH levels.

The Antioxidant Response Element (ARE) is a cys-acting transcriptional regulatory element implicated in the expression of antioxidant and cytoprotective proteins. NF-E2-related factor 2 (Nrf2) is a transcriptional factor, responsible for the constitutive and inducible expression of ARE. Under basal physiological conditions, Nrf2 is kept low by proteasomal degradation, which is induced by constitutive ubiquitination via the adaptor protein Kelch-like erythroid cell-derived protein-1 (Keap-1). Under redox-imbalance conditions, Keap-1 undergoes conformational changes and frees Nrf2 to translocate to the nucleus and activate ARE-regulated genes [76,77,78]. To restore redox balance and encourage cell survival, a number of antioxidants, antiapoptotic, metabolic, and detoxifying proteins are activated when Nrf2 binds to ARE [79]. Experimental UVB effect on Nrf2 varies depending on radiation intensity, cell type, and time-lapse, although in most of the studies, UVB tends to show negative effects on Nrf2 expression and activity [78]. According to experimental data, Nrf2 knockout mice typically exhibit a greater inflammatory response and overexpression of several inflammation markers compared to wild-type mice, such as pro-MMP-9 and p53, a regulator of DNA repair, indicating that the absence of Nrf2 results in more sustained DNA damage [80].

In the present work, UVB promoted a negative effect on Nrf2 expression, but AT-RvD1 enhanced the mRNA expression of Nrf2 compared to the UVB control group, suggesting that the protective results observed with AT-RvD1 treatment could be explained by upregulation of the Nrf2 pathway. Through this same mechanism, AT-RvD1 has previously shown pro-resolution activity. The aforementioned study was performed employing a murine model of lung emphysema induced by cigar smoke, and AT-RvD1 was capable of decreasing the levels of ROS, neutrophils, MMP3 and MPO activity as well as increasing macrophage counts and IL-10 levels [15]. Accordingly, our results also show upregulation of NAD(P)H quinone-oxidoreductase 1 (NQO-1) in samples from mice treated with AT-RvD1 when compared with the UVB-stimulated group. NQO-1 is a downstream enzyme regulated by Nrf2, along with other enzymes including heme-oxygenase 1(HO-1), glutamate-cysteine ligase (GCL), glutathione S-transferases (GSTs), superoxide dismutase (SOD), thioredoxin UDP-glucuronosyltransferase, and catalase [81,82]. Catalase plays an important role in lipoperoxidation prevention because it catalyzes the conversion of H_2_O_2_ into water, preventing the formation of HO^•^ [83]. It has been established that UVB radiation causes catalase depletion [57]; nevertheless, therapy with AT-RvD1 could restore catalase activity in the present study, reinforcing AT-RvD1’s protective actions in maintaining the epidermis’s normal redox balance. Other studies employing the same mouse model of UVB-induced skin damage have shown similar effects on the expression of Nrf2 and related enzymes including NQO-1 and catalase, supporting the notion that AT-RvD1 protective mechanism in this model also involves activation of the Nrf2 pathway [23,26,83]. Our results suggest that by upregulating the Nrf2 pathway, AT-RvD1 promotes the expression of ARE genes, restoring the natural antioxidant defense in UVB-exposed skin to avoid oxidative stress and promote the resolution of inflammation.

Despite their powerful biological actions, SPMs are expected to have no significant side effects likely because they are endogenous molecules that trigger physiological reparative responses, which seem to be events to prevent disease progression. The mechanisms activated by SPMs may be accountable for this absence of adverse effects. In fact, SPMs do not act solely by inhibiting inflammatory mechanisms and cells but regulate the immune system and stromal cells to produce fewer inflammatory molecules and upregulate the production of protective mediators with pro-resolution actions by shifting their cellular profile [84,85]. It has been demonstrated that the D series resolvins did not evoke immunosuppression [86] or tumor growth [87]. Taking cancer as an example to be further detailed, RvD1 or AT-RvD1 have been shown to reduce the progression of hepatitis to liver cancer [88], and the proliferation of hepatoblastoma and PLC/PRF/5 hepatocellular carcinoma [89]. RvD1 or AT-RvD1 also reduced the epithelial-to-mesenchymal transition, which is considered an initiation event in tumor metastasis [90], thus aligning with, for instance, the observation that RvD1 inhibited lung cancer progression and metastasis [91]. RvD1 and AT-RvD1 among other SPMs and AT-SPMs could inhibit debris-stimulated cancer progression, an effect that was explained by the enhancement of debris phagocytosis by macrophage in multiple tumor types [92,93]. In line with the effect of AT-RvD1 in immune cells that protect from cancer, this SPM reduced the ration of tumor-associated neutrophils/lymphocytes in a lung adenocarcinoma model, thus reducing tumor growth [87]. Genetic deletion of 15-lypoxygenase or of the RvD1 receptor GPR32 in gastric cancer cells led to an increase in the angiogenic and tumorigenic activities of those cells, therefore, demonstrating a protective role of those endogenous pathways involved in SPM production and action [94]. Alternatively, there are also studies that did not find evidence of the participation of RvD1 or AT-RvD1 in cancer. For instance, no association was found between the occurrence of adenoma and plasma levels of RvD1 [94]. Although AT-RvD1 reduces the lung inflammation and pathology caused by chronic dust, it does not alter the susceptibility to dust-enhancement of carcinogenesis [95]. There is also one study which found that RvD1 plasma levels are increased in pancreatic cancer patients compared to healthy volunteers, which was independent of the cancer stage [96]. This study identified RvD1 as a plasma marker of disease although the role of RvD1 was not investigated. These examples lead us to conclude that AT-RvD1 and RvD1 have mostly positive therapeutic actions or endogenous roles in varied types of cancer, and sometimes, no role at all. However, whether RvD1 is involved in pancreatic cancer development or is an endogenous mechanism aimed to reduce tumor growth remains to be determined [96]. Maybe RvD1 can have a different role from what is expected depending on the cancer type, which merits further investigation to prevent potential side effects of RvD1 or AT-RvD1 treatment.

Supporting a potential therapeutic application of SPMs, in a phase III clinical trial, the RvE1 synthetic analog significantly reduced the signs and symptoms of dry eye syndrome without side effects, marking the first evidence of pharmacological action of an isolated SPM’s clinical efficacy. The AT-LXA4 analog, 15-R/S-methyl-LXA4, reduced infantile eczema with no apparent toxicity or side effects [97,98]. Nevertheless, because of the great potential for clinical use, further research is necessary to better characterize the safety of isolated AT-RvD1 by assessing adverse effects, tolerability, and pharmacological interactions.

## 4. Materials and Methods

### 4.1. Chemicals

The 2,2′ azino-bis (3-ethylbenzothiazoline-6-sulfonic acid) (ABTS+), *2*,*4*,*6*-*Tripyridyl-s-triazine* (TPTZ), 5,5’-dithiobis-(2-nitrobenzoic acid) (DTNB), bisacrylamide, hexadecyl trimethyl ammonium bromide (HTAB), brilliant blue stain, phenanthroline, phenylmethylsulfonyl fluoride, reduced glutathione (GSH), nitroblue tetrazolium (NBT), o-dianisidine dihydrochloride, and Trolox were obtained from Sigma-Aldrich (St. Louis, MO, USA). Tris was obtained from Amresco (Solon, OH, USA). Enzyme-linked immunosorbent assay (ELISA) kits were obtained from eBioscience (San Diego, CA, USA). Acrylamide sodium dodecyl sulfate (SDS), Superscript1III, Oligo(dT)12-18 primers, Platinum SYBRGreen I, and primers were obtained from Invitrogen (Carlsbad, CA, USA). AT-RvD1 was purchased from Cayman Chemicals (Ann Arbor, MI, USA). All other reagents used were of pharmaceutical grade.

### 4.2. Animals and Experimental Protocol

Experiments were performed using female, 20–30 g hairless mice (HRS/J) randomly assigned to five groups of five animals each, labeled as follows:

(1) Naive: group not exposed to UVB radiation.
(2) UVB: group stimulated by UVB radiation and treated with the vehicle.
(3) 30: group stimulated by UVB radiation and treated with 30 pg/animal AT-RvD1.
(4) 100: group stimulated by UVB radiation and treated with 100 pg/animal AT-RvD1.
(5) 300: group stimulated by UVB radiation and treated with 300 pg/animal AT-RvD1.

All groups except naive were exposed to 4.14 J/cm^2^ UVB simultaneously [31]. Treatments consisted of iv retro-orbital injections of AT-RvD1 dilutions in saline or saline-only (UVB-stimulation group) 30 min before radiation exposure. After terminal anesthesia with isoflurane (5% in O_2_), samples of the skin were collected at three different time points considering the alterations that occur during the course of UVB skin inflammation. At 12 h after UVB radiation, samples were collected for edema; GSH level; FRAP and ABTS scavenging antioxidant capacity; myeloperoxidase (MOP) and metalloproteinase-9 (MMP-9) activities; and histopathological analysis for epidermal thickness, sunburn keratinocytes, dermal mast cells, and collagen degradation. At 4 h after UVB radiation, samples were collected for cytokine production and COX-2 mRNA expression levels by RT-qPCR. At 2 h after UVB radiation, samples were collected for catalase activity and O_2_^•−^ production. Samples for edema were weighed immediately after the collection, samples for histology were kept in a 10% paraformaldehyde solution at RT until analysis, and samples for the remaining tests were stored at −70 °C until analysis. These protocols and time points of sample collection were previously standardized in our laboratory [19]. Unless otherwise stated (see the detailed description of each assay below), skin samples from each animal (about 30 mg for assay) were collected in the appropriate solution, weighed for calculations and triturated with a tissue-tearor at ice temperature. The Animal Ethics Committee of the Londrina State University (CEUA—UEL) approved this study under the process number 1447.2015.10 on 19 March 2015. The total number of mice was 152 and number of mice per type of analysis was mentioned in each figure caption.

#### 4.2.1. Skin Edema

Round skin samples were cut with a standard-sized puncher and weighed immediately after the collection. The weights from treated groups (10, 30, and 300 pg/animal AT-RvD1) were compared to those from naive and UVB groups. All the results were expressed in mg of skin [30].

#### 4.2.2. Neutrophil Infiltration

Myeloperoxidase (MPO) is an enzyme extensively expressed by neutrophils; therefore, it is considered a suitable marker for these cells’ presence. MPO can promote the oxidation of o-dianisidine to a colored compound, leading to an increase in absorption [34]. Skin samples were collected and triturated in 0.05 M (pH 6.0) phosphate buffer with 0.5% hexadecyl trimethyl ammonium bromide (HTAB) and centrifuged (16,600× *g*; 4 °C; 2 min). A supernatant aliquot was mixed with an o-dionisidine solution (0.167 mg/mL) and hydrogen peroxide (0.015%). The spectrophotometric readings were taken at 450 nm. The results (neutrophils/mg of skin) were calculated using a neutrophil concentration curve [21].

#### 4.2.3. Cytokine Production

Samples were collected and triturated in sterile saline solution and centrifuged (2000× g, 4 °C, 15 min). Aliquots of the supernatants were analyzed using Enzyme-Linked Immunosorbent Assay (ELISA), following the manufacturer’s (eBioscience) guidelines. The results were expressed in picograms (pg) of each cytokine/mg [30].

#### 4.2.4. Histopathological Analysis of the Skin

Skin samples were prepared following standard protocols (fixed in 4% paraformaldehyde, dehydrated in ascending ethanol concentrations, cleared in xylene, and embedded in paraffin). The specimens were cut into 5 μm thick slices and stained according to the analysis requirements:

- For infiltration of mast cells in the dermis: Specimens were stained with Toluidine Blue. Cell counting was performed using the software Infinity Analyze 6,5 (Lumenera, OT, Canada) [99].
- For epidermal thickness [100] and the number of sunburn cells [101] determinations: specimens were stained with hematoxylin and eosin (HE). For epidermal thickness, measures were made using the software Infinity Analyzer (Lumenera R Software).
- For collagen fiber density determination: Specimens were stained with Masson’s Trichrome. The blue color intensity of the dermis was proportional to the collagen density.

Naive and UVB groups were compared to treated groups (10, 30, and 300 pg/animal 17(R)-RvD1) using Image J software (NIH) [102].

#### 4.2.5. mRNA Expression for COX-2, Nrf2, and NQO-1

Samples were collected, finely chopped, and immersed in Trizol for RNA extraction. The expression of mRNA for COX-2, Nrf2, and NQO-1 was determined by reverse transcriptase and quantitative polymerase chain reaction (RT-qPCR) using the GoTaq12-Step RT-qPCR System (Promega) on a Step One Plus TM Real-Time PCR System (Applied Biosystems1). The expression of ꞵ-actin mRNA was used as a control for tissue integrity in all samples [23].

The primers used are specified in Table 1.

#### 4.2.6. Metalloproteinase-9 (MMP-9)

The samples were triturated with the aid of a tissue-tearor homogenizer, using a ratio of 1 g of skin to 4 mL of a 50 mM (pH 7) solution of Tris/HCl with calcium chloride (CaCl_2_) and 1% of protease inhibitors. The homogenates were centrifuged twice at 12,000× *g* for 10 min at 4 °C, and the supernatants were analyzed using zymography in polyacrylamide sodium with added dodecyl sulfate (SDS) and gelatin. The assay is based on the detection of gelatin degradation by MMP-9, which is correlated to the intensity of band discoloration against the dark background of the Coomassie-Blue-stained gel. The proteolytic activity was quantified by the comparison of data from treated (10, 30, and 300 pg/animal AT-RvD1) and control groups (naive and UVB) using ImageJ^®^ software (NIH, Bethesda, MD, USA) [26].

#### 4.2.7. FRAP and ABTS Assays

FRAP capacity and ABTS scavenging activity were measured in aliquots of skin collected and triturated in 1.15% KCl solution, according to Casagrande et al. (2006). The results were compared to a Trolox standard curve (0.01–20 nmol) and presented as nmol Trolox equivalent per mg of skin [21].

#### 4.2.8. GSH Assay

The GSH sulfhydryl group promotes 5,5’-dithiobis-(2-nitrobenzoic acid) (DTNB) breakage of the disulfide bond, causing the appearance of a yellow compound (5-mercapto-2-nitrobenzoic acid), which can be colorimetrically quantified. Each sample (about 100 mg) was collected, triturated, and homogenized in a 0.02 M EDTA solution (in the rate of 1 g skin for 4 mL of solution). Homogenates were treated with 50% trichloroacetic acid and centrifuged twice (2700× g, 4 °C, 10 min), and the supernatants were used to test for GSH levels. The data were analyzed using a standard curve of GSH (5–150 μM), and the results were presented as μM GSH per mg of skin [103].

#### 4.2.9. Catalase Assay

Each sample was collected and triturated in 0.02 M EDTA solution and centrifuged twice (2700× *g*, 4 °C, 10 min). Catalase activity was based on UV absorbance decay due to H2O2 degradation and the generation of oxygen. The results were obtained by the difference between the initial reading (at 240 nm) and a second reading at 30 s after H_2_O_2_ addition. The results were expressed as units of catalase/mg of skin/minute.

#### 4.2.10. Superoxide Anion (O_2_^•−^) Production

O_2_^•−^ can reduce NBT to a dark, purple-colored compound known as formazan, measured by optical density. Samples were collected, triturated in 0.02 M EDTA, and centrifuged (2000× *g*, 4 °C, 20 s). An aliquot of the supernatant was incubated with (NBT 1mg/mL), and formazan was dissolved with KOH 2 M and dimethylsulfoxide and measured at 620 nm. The results were expressed OD/10 mg of skin [31].

#### 4.2.11. Statistical Analysis

Statistical analysis was performed using GraphPad Prism software version 7 (GraphPad Software Inc., San Diego, CA, USA). Data were analyzed by one-way analysis of variance (ANOVA) followed by the Tukey multiple comparisons test. The results present the mean ± standard error (SEM) of measurements made with 5 animals per group per experiment and are representative of 2 separate experiments and were considered significantly different at *p* < 0.05.

## 5. Conclusions

In the same model used in the present study, RvD1 is active at 30 ng/mouse dose to reduce skin inflammation and oxidative stress caused by UVB. AT-RvD1 was active at the dose of 300 pg/mouse. This 100-fold dose difference suggests that AT-RvD1 is a promising resolvin to be used as a drug. The results showed that AT-RvD1 treatment reduces biochemical, immune, and histopathological changes occurring in the skin upon UVB radiation. This beneficial effect of AT-RvD1 seems to be dependent on inducing Nrf2 since this is a master transcription factor that upregulates antioxidant responses and reduces inflammation by the expression of its target genes. Right now, the route of administration used to administrate AT-RvD1 (i.v.) is an issue and developing orally active forms to deliver AT-RvD1 might be an essential step to allow translating these results into clinical settings.

## Figures and Tables

**Table 1:** Primers used to perform mRNA expression determinations by RT-qPCR.

Primer	Sense	Antisense
ꞵ-actin	5′-CGGTTCCGATGCCCTGAGGCTCTT-3′	5′-CGTCACACTTCATGATGGAATTGA-3′
COX-2	5′-AACCGCATTGCCTCTGAAT-3′	5′–CATGTTCCAGGAGGATGGAG-3′
Nrf2	5′-TCACACGAGATGAGCTTAGGGCAA-3′	5′-TCACACGAGATGAGCTTAGGGCAA-3′
NQO-1	5′-TGGCCGAACACAAGAAGCTG-3′	5′-GCTACGAGCACTCTCTCAAACC-3′

## Data Availability

The data that support the findings of this study are available from the corresponding author upon reasonable request.

## Sample Availability

All compounds are available for purchase from the mentioned companies.
